# A way forward for the South African quail sector as a potential contributor to food and nutrition security following the aftermath of COVID-19: a review

**DOI:** 10.1186/s40066-021-00331-8

**Published:** 2021-12-09

**Authors:** C. M. Mnisi, M. Marareni, F. Manyeula, M. J. Madibana

**Affiliations:** 1 Department of Animal Science, Faculty of Natural and Agricultural Sciences, North-West University, P Bag x2046, Mmabatho, 2735 South Africa; 2 Food Security and Safety Niche Area, Faculty of Natural and Agricultural Science, North-West University, Mafikeng, South Africa; 3 Faculty of Animal and Veterinary Sciences, Department of Animal Sciences, Botswana University of Agriculture and Natural Resources, Gaborone, Botswana; 4 Department of Forestry, Fisheries and the Environment, Marine Research Aquarium, Lower Beach Road, Sea Point, South Africa

**Keywords:** Farmers, Feed sources, Poultry consumers, Markets, Natural additives, Quail

## Abstract

Commercial quail (*Coturnix coturnix*) farming has recently gained recognition from the South African poultry industry as a potential source of protein, which can be used to alleviate protein-energy malnutrition as well as food and nutrition insecurity in rural South Africa. Over six large-scale and hundred small-scale farmers are currently producing various quail breeds for commercial purposes in South Africa. However, these farmers face challenges of high feed costs, diseases, poor health care, low demand (for quail meat and eggs) and limited access to the markets. In addition, the lack of a pre-existing local market for quail meat and eggs has seen most farmers exporting these products to other countries, but with low profit margins owing to the slow growth experienced by world economies. Furthermore, the socio-economic crisis brought by the global Coronavirus Disease 2019 (COVID-19) pandemic has exacerbated these challenges as most of the countries enforced nationwide lockdown to mitigate the spread of the virus. Although this initiative was taken to save lives, it left quail farmers not knowing where to trade their stock due to large uncertainties in the social and economic domain, compounded by the fact that many South African citizens are traditionally accustomed to chicken products. Moreover, the size of the quail in relation to its market price when compared to that of chickens continues to lower its demand causing a lot of quail businesses to collapse. Thus, it is imperative to explore strategies that can reduce the cost of producing quail, while increasing their demand and popularity. Non-conventional feed ingredients and phytogenic feed additives that are inexpensive, locally available, and readily accessible should be identified and evaluated in quail diets to deliver sustainable production systems that will ensure that these birds continue to play a significant role in food and nutrition security of humans. In addition, extension service workers and quail farmers need to form a collaborative team to increase awareness about the benefits of quail products and build a long-lasting and profitable quail business. In this work, we present potential rearing methods for commercial quail production, nutritional benefits of quail products, as well as nutritional solutions for a sustainable and profitable quail business. Lastly, we review prospective awareness programs and marketing strategies that are aimed at successful commercialisation of quail using various networks.

## Introduction

The whole world has recently seen the outbreak of a deadly virus known as Coronavirus Disease 2019 (COVID-19) pandemic, which has devastated the economies and livelihoods of both the developed and developing countries [1]. As an attempt to contain the spread of this fatal virus, most governments have enforced nationwide lockdown in their respective countries [2, 3], including South Africa whose economy had just been downgraded to a low investment grade by the Moody’s Investors Service in the first quarter of 2020 [4]. Although these measures were put in place to save lives and reduce transmissions from one person to another, many people lost jobs, businesses, and livelihoods, which also saw a significant rise in unemployment [5]. The nationwide lockdown meant that the general citizenry would not be able to make ends meet to maintain their living requirements, as such, poverty (hunger and malnutrition) became another noticeable pandemic [6, 7]. Without a doubt, this virus has exacerbated issues of food and nutrition insecurity forcing many governments to put in place relief measures as a temporary strategy to combat social ills [8].

Even though the agricultural sector was given a green card as an ‘essential sector’ to operate during the pandemic [3], many farmers found themselves in a predicament of not knowing where to trade their stock given that both national and international markets were closed, and most countries had imposed travel bans [9]. This meant that small–medium business enterprises, particularly quail (*Coturnix coturnix*) farmers, could not continue with business as usual or even export their produce to foreign markets; and because quail demand is very low in South Africa, most quail businesses were negatively affected. The aftermath of this virus would certainly last for decades, hence there is an urgent need to identify and explore various strategies that would ensure a sustainable and profitable quail business that continues to contribute towards food and nutrition security.

In South Africa, there are over six large-scale and hundred small-scale quail producers who are registered in the national database, and many other unregistered emerging farmers who trade with local communities [10]. The best performing quail farmer produces nearly 16,000 birds per production cycle, and local demand for quail meat was estimated to be 48 tons in 2016 [10]. On average, one commercial quail farm in South Africa markets around 435 kg of eggs per year [10]. Quail meat and eggs are rich sources of high-quality protein, polyunsaturated fatty acids, minerals, and vitamins [11, 12] that could be essential towards eradicating malnutrition in South Africa. In addition, quail eggs are reported to improve vision, boost body energy levels, stimulate growth by enhancing metabolism, reduce blood pressure, cleanse the human body, and prevent chronic diseases [12, 13]. In other reports, regular consumption of quail eggs reduces stomach ulcer, boost the immune system, promote memory health, increase brain activity, and stabilise the nervous system [14, 15]. Unfortunately, quail birds are not very popular in South Africa, and consequently remains excluded from the markets [14].

The major challenge in the long-term sustainability of intensive quail production is high feed cost, which amounts to nearly 70% of the total costs of production [16]. This is caused by the escalating prices of conventional feed ingredients such as maize and soybean, which is due to their high demand by the biofuel, food, and animal feed sectors [17, 18]. This suggests that a lot still must be done, especially from production to market to revitalise the quail business. This work reviews potential rearing methods and nutritional solutions that can be used to optimise sustainable quail production systems that are based on high welfare standards to alleviate protein-energy malnutrition and food insecurity in South Africa. In addition, it reviews practical interventions and economic strategies that can be employed to increase the popularity and demand of quail, while building a long-lasting local market.

## Potential rearing methods for commercial quail production

Quail is a collective term for small-sized birds that belong to the *Galliformes* order. There are over hundred breeds of quail in the world, which are divided into two groups, namely: New World and Old World quail [11, 19]. The New World quail belongs to the *Odontophoridae* family, whereas the Old World quail belongs to the *Phasianidae* family [19]. The *Coturnix coturnix* sp. is the most popular quail breed in South Africa. The *Coturnix* strain was first domesticated in Japan and later introduced in America, Europe, and the Middle East between 1930 and 1950 [11, 19]. To date, China, Spain, France, Italy, Brazil, USA, and Japan are leading countries in commercial quail production [20]. Quail rearing can occur under different management and production systems, which depend on the breed type, input and output level, purpose of production, and flock size [21]. For large-scale production, cages with wire-mesh floors are used so that waste can easily fall through into a collection area [11]. In South Africa, the deep litter and battery cage systems are commonly used for quail production. In a battery cage system, quail can be reared in spaces of 75, 150 and 175 cm^2^/bird at ages of 0–4, 5–6 and > 6 weeks, respectively [22]. However, in a deep litter system, the spaces recommended for quail at 0–4, 5–6 and > 6 weeks of age are 75,200 and 250 cm^2^/bird, respectively. Nonetheless, Padmakumar et al. [23] argued that rearing systems and different floor densities (100, 150, 200 and 250 cm^2^/bird) do not influence liveability. Razee et al. [24] reported that quail perform optimally in a battery cage system because cages limit flight mode and improve their management. The authors further recommended the use of either the battery cage system or the deep litter system on floor density of 200 cm^2^/layer quail. However, there are limited studies that investigated the optimum stocking density and welfare of both layer and broiler quail under the two rearing systems. Comparing the two systems, the construction of floor for deep litter is easy and affordable compared to buying cages for the battery cage system.

### Improving quail welfare and health care

Like in any other farming enterprise, strict biosecurity measures are important to prevent cross-contamination and disease outbreaks, while ensuring the production of healthy products that are safe for human consumption. Although often overlooked, these measures involve controlled access to the farm, shower-in and shower-out, regular cleaning and removal of bedding material, and disinfection and fumigation, which start with the handling and rearing of the birds at farm level and ends at slaughter point. Several animal rights committees have been formed to monitor the welfare and care of farm animals, but there is no specific committee for quail rearing and management in South Africa. The quail is naturally a wild-game bird that even when domesticated, it must be allowed to exhibit its natural traits as it would in its natural habitat. In addition, the quail should always have free access to feed and clean water [25]. Valencia [21] reported that for a quail to attain optimal performance, a standard game-bird ration must be fed because it has a high nutritional value, especially protein, to emulate their natural diet. Indeed, wild quail birds feed on a variety of insects that have a high biological value [26]. Generally, a quail has strong immunity and resistance against several poultry diseases [27], which indicates a possibility for limited usage of antibiotics or vaccines in their production. However, veterinary training should be a prerequisite for all farmers to keep a healthy flock and allow the detection of abnormal conditions (blindness, twisted legs and/or necks) and other common illnesses such as inflammations, enteritis, coccidiosis, etc. Quail are social birds that should not be housed in isolation, but in groups. As such, the grouping of quail birds should consider their gender, age and stage of production to avoid cannibalism, a common behaviour in a quail flock.

## An overview of the nutritional benefits of quail products

### Quail meat

Quail produces a tasty and delicate white-game meat that is rich in essential nutrients such as protein, vitamins, and minerals (calcium, potassium, phosphorous, iron and zinc), and has low skin fat and cholesterol content [28, 29]. Quail meat has higher monounsaturated fats than chicken meat, which is good for human health and growth [22, 30]. The ideal protein for a human being should contain lysine (5.5%), leucine (7%), isoleucine (4%), valine (5%), methionine + cysteine (3.5%), threonine (4%), phenylalanine + tyrosine (6%) [31]. Thus, quail meat can be an essential source of dietary amino acids for humans as indicated in Table 1. Thus, a deficiency in dietary protein due to a lack of one of these amino acids could lead to impaired immune function and increase the vulnerability of human to infectious diseases [32]. Africa has the lowest protein intake per capita per day when compared to the other continents [33], thus the reliance on quail meat as an alternative protein source will certainly grow in the nearest future, especially for people in the global South.

**Table 1:** Nutritional composition (%) of quail meat

Nutrients	Sources			
	[37]	[28]	[30]	[38]
Proximate compositions				
Moisture	72.59	72.39	73.50	74.59
Protein	23.38	21.65	18.4	22.82
Fat	2.21	3.57	4.21	1.77
Ash	1.51	2.47	0.94	1.24
Amino acids compositions				
Lysine	2.19	8.99	*	7.12
Methionine	0.56	2.69	*	3.18
Isoleucine	1.22	5.10	*	5.37
Leucine	2.69	8.15	*	7.44
Phenylalanine	0.97	4.68	*	5.48
Threonine	0.74	4.58	*	6.02
Valine	1.29	5.20	*	7.12
Cysteine	0.20	1.34	*	0.87
Tyrosine	0.61	3.49	*	2.85

* = not reported

Quail meat contains essential fatty acids required by the human body as an energy source in complex and interconnected systems, structure for cell membrane, modulate gene transcription and as a function in cytokines precursors [34]. As such, the consumption of quail meat can improve human health and reduce malnutrition associated with energy deficiency. Quail meat contains fat content ranging from 1.58 to 4.21% and a cholesterol content ranging between 62.3 and 68.2% [35]. From a medical viewpoint, high fat and cholesterol contents can cause high blood pressure and heart related diseases in humans [36], a view that led to consumers to be cognizant of the food they consume. Thus, when compared to chicken meat, quail meat has the lowest fats and cholesterol contents which is desirable for health-conscious consumers [35].

Quail meat is a good source of oleic, linoleic, palmitic, and stearic acids (Table 2) and the net composition of fatty acids is 87.8 and 89.2% for breast meat in young and spent quail, respectively [28]. Genchev et al. [37] also reported high oleic, palmitic, linoleic, and stearic acids in quail meat that amounts up to 88.3% in breast meat and 88.3% in leg meat of total lipids [39]. Oleic is known to improve lipid profiles, balance of body weight and skeletal muscle [40]. It is increasingly apparent that the consumption of quail meat can improve these vital functions and subsequently boost human health. Compared to other poultry meats, quail meat can be used as a source of immune-supportive nutrients to boost human health [29], particularly in a time when the entire world is battling against the negative effects of COVID-19 pandemic.

**Table 2:** Fatty acid contents (%) of quail meat

Fatty acids	Sources			
	[37]	[41]	[28]	[42]
Myristic (C14:0)	0.95	0.74	0.70	0.95
Myristoleic (C14:1)	*	*	*	1.15
Palmitic (C16:0)	24.39	25.76	19.81	20.42
Palmitoleic (C16:1)	*	*	3.85	0.46
Stearic acids (C18:0)	8.75	7.49	6.27	5.00
Linoleic (C18:2)	*	*	28.85	23.62
Arachidic acids (20:0)	*	0.36	0.65	*
Behenic acids (22:0)	*	*	0.83	*
ƩSFA	34.13	34.65	27.82	26.37
ƩUFA	65.68	*	76.01	*
ƩMUFA	40.70	*	44.55	36.61
ƩPUFA	24.98	*	30.66	36.21

* = not reported

### Quail eggs

A quail egg is an excellent source of nutrients (Table 3) such as protein, lipids, vitamins (A, C, D and B complex) and minerals as well as other substances such as lysozymes, ovomucoid and cystatin that have therapeutic effects [12, 13]. The protein quality of a quail egg is characterised by high leucine, lysine, and valine contents [12, 42], which are required for normal functioning of the body. For example, lysine is required to produce antibodies, hormones and enzymes as well as assisting in calcium absorption [32]. Leucine regulates blood sugar level and maintains a balance of insulin and glucose, while valine repairs of body tissues, muscle growth and metabolism [32]. Furthermore, quail eggs contain non-essential amino acids such as aspartic acid, serine, and alanine, which are important for forming several compounds that are involved in metabolic processes in the human body [14, 15].

**Table 3:** Nutritional composition of quail eggs (%, unless otherwise stated)

Nutrients	Sources				
	[43]	[30]	[14]	[12]	[44]
Proximate composition					
Moisture	70.9	71.0	*	72.9	*
Protein	13.3	16.3	13.7	12.3	13.1
Fats	0.63	2.6	11.9	11.4	11.1
Ash	1.1	1.1	1.0	1.1	*
Fibre	*	*	0.6	*	*
Carbohydrates	1.6	9.1	*	1.64	0.4
Cholesterol	12.3	*	*	12.26	*
Mineral and vitamin composition (mg/100 g)					
Calcium	*	149.1	*	31.45	*
Iron	*	2.06	*	3.01	*
Zinc	*	1.28	*	3.16	*
Vitamins A	*	*	*	0.36	*
Vitamins E	*	*	*	5.9	*
Vitamins B6	*	*	*	0.65	*
Vitamins D	*	*	*	0.45	*

* = not reported

Phosphorus, nitrogen, potassium, calcium, magnesium, copper, sodium, zinc, and iron are principal elements in whole quail eggs [13, 45] and are used by the body to perform functions that are crucial in life. For example, iron is essential for impairment of oxidative burst and bacterial killing by enhancing the proliferation of T-lymphocytes [46], while zinc plays a vital role in maintaining good health. Read et al. [47] reported that zinc inhibits RNA viral (COVID-19) replication process by inactivating RNA polymerase. However, the quality of a quail egg can be affected by factors such as stocking density, feed composition, bird’s age, storage time and environmental conditions [48]. As such, sustainable intensification of layer quail would promote continuous supply of essential nutrients, while reducing the prevalence of nutrient deficiency, especially in rural areas of South Africa.

The human body requires two types of fatty acids viz., linoleic, and α-linolenic acids [34] because of the lack of desaturases enzymes necessary for their synthesis. Quail eggs are rich in linoleic acids (Table 4) and can be an unequivocal source [12]. Interestingly, quail eggs have high polyunsaturated fatty acids (PUFA) and low saturated fatty acids (SFA). Previous reports indicated that saturated fatty acids increase the levels of low-density lipoprotein cholesterol, which contributes to heart diseases [49].

**Table 4:** Fatty acids contents (%) of quail eggs

Fatty acids	Sources			
	[12]	[42]	[44]	[30]
Myristic (C14:0)	0.94	0.79	0.6	0.32
Myristoleic (C14:1)	*	6.29	*	*
Palmitic (C16:0)	27.53	28.11	25.2	21.6
Palmitoleic (C16:1)	4.85	2.60	6.3	6.66
Stearic acids (C18:0)	5.34	8.62	7.7	9.0
Linoleic (C18:2)	16.61	15.36	10.2	12.05
Arachidic acids (20:0)	0.08	*	*	
ƩSFA	34.07	37.52	*	41.4
ƩUFA	*	*	*	
ƩMUFA	47.07	54.12	*	36.7
ƩPUFA	18.46	8.36	*	21.9

* = not reported

## Nutritional strategies to promote a sustainable and profitable quail business

### Use of potential protein sources

Protein quality is measured by its ability to supply sufficient quantities of essential amino acids [50]. Fast-growing quail birds require high amount of dietary protein (24%) to fully express their genetic potential [51]. To meet this requirement, soybean meal, a protein-rich vegetable source, is widely used during diet formulations. Soybean meal has high crude protein content that is composed of highly digestible amino acids [52]. Soybean is a principal protein source for intensive poultry production and is regarded as a’gold standard’ protein source to which all other protein ingredients are compared [18, 50]. However, continuous reliance on soybean meal for commercial quail production is unsustainable due to its high demand by the food, biofuel, and animal sectors [18], as well as prevailing high market prices coupled with declining soy production because of unfavourable climatic conditions [53]. Thus, sustainable quail production in tropical countries such as South Africa would require the use of alternative protein sources that can acclimatise to erratic rainfall patterns, scorching heat with prolonged drought period, and poor soil fertility [54].

### Alternative plant protein sources

#### Canola

Canola is a rapeseed biotype that has been developed using plant breeding methods to minimise the toxic glucosinolate content [55]. Rapeseed plant breeders used traditional breeding methods to remove a significant portion of erucic acid and glucosinolates from flowers, resulting in a drastic rise in rapeseed production in Europe and Canada [55]. The term canola was later adopted in Canada to describe a crop with low levels of both compounds and high nutritive value [56]. Canola meal is an oil seed meal with a protein-rich concentration and a well-balanced amino acid profile that has been proposed as a suitable alternative to soybean meal in poultry diets [55]. The amino acid profile of canola and soybean meal is comparable, but with noted variations in lysine (1.72 and 2.57%) and methionine contents (0.70 and 0.50%), respectively [57]. Canola meal has no direct food value for humans and can be procured at a relatively lower cost than soybean meal. Consequently, the use of canola meal in quail diets could allow versatility in feed formulation. Several studies have reported that the inclusion of canola meal up to 120 g/kg had no negative influence on feed utilisation, performance, and meat quality traits of the quail [16, 58].

#### Moringa

*Moringa oleifera* is a deciduous perennial plant native to India and has since spread to other parts of the world including South Africa [59]. The rapid propagation of moringa can be attributed to its ability to yield high biomass in a wide range of environmental conditions. In addition, its tuberous tap root enhances its ability to withstand drought conditions of semi-arid regions [54]. The leaves are the most nutrient-dense component, providing biocompounds such protein, amino acids, carbohydrate, minerals, organic acids, flavonoids, and vitamins (B, C and K) as well as provitamin A in the form of beta-carotene [60]. Moyo et al. [61] reported a protein content of 30.29% with a well-balanced amino acid profile for the South African biotype. Supplementing Japanese quail diets with *Moringa oleifera* powder at a rate of 1–3 g/kg was shown to improve egg production and hatchability, as well as enhancing egg quality traits [62, 63].

#### Mucuna pruriens

Velvet bean (*Mucuna pruriens*) is a tropical underutilised legume with high biomass production of foliage and seeds, which is predominantly found in Asia and Africa [64]. Velvet bean contains a substantial protein content (31.75–35.5%), which is composed of amino acids that are similar to that of soybean [65]. However, the value of velvet bean as food for humans or animals is scanty because it has been grown as a cover crop to protect and replenish soil fertility. In addition, the utility of velvet bean as a protein source in quail diets may be hindered by high levels of anti-nutritional factors such as levodopa, trypsin inhibitors, oxalate, alkaloids, cyanogen, lectins and phytate [66]. Accordingly, pre-processing by soaking, autoclaving, irradiation, thermal and enzyme treatment can be used to improve the feed value of the beans [67]. Indeed, the inclusion of toasted mucuna seed meal up to 15% in quail layer diets was reported to improve performance and egg quality parameters of the hens [67].

#### Marama

Marama (*Tylosema esculentum*) bean is a wild-growing perennial legume that is native to the Kalahari Desert and surrounding areas in Botswana, Namibia, and northern regions (Northern Cape, North-West, Gauteng, and Limpopo) of South Africa [68]. Marama bean is a valuable protein source with a high composition of protein (29–38%) and lipids (32–42%) [69]. It has a considerable amount of trypsin inhibitors [70], which may negatively affect protein utilisation in quail diets. To date, there are no documented studies that have evaluated the feed value of this novel protein source in quail diets. However, thermal processing of marama bean with dry heat was shown to improve protein quality and reduce its fat content, possibly due to the disruption of lipid bodies during dry heating [71].

### Alternative animal protein sources

The latest research into the large-scale production of insect proteins for aquaculture and poultry is driven by the desire to use inexpensive protein sources with low carbon footprint but high biological value [18]. The increasing demand for insect-based feed products has led to the establishment of a new market niche with a great potential to replace fishmeal and soybean meal, which are currently the main protein sources used in animal feeds [72]. A variety of insects, which include grasshoppers, crickets, cockroaches, termites, lice, stink bugs, cicadas, aphids, scale insects, psyllids, beetles, caterpillars, flies, fleas, bees, wasps, and ants have been incorporated in poultry diets in fresh, dry and mash form as protein supplements [26, 73, 74]. Industrial mass production of various insects has been carried out in some parts of world including South Africa, which includes the use of reproduction trays where the adult insects would mate and lay eggs. The produced larva is then grown in a medium that is enriched with the insect’s substrate until it is harvested, dried, and milled to be incorporated in animal rations [72]. Among these insects, the black soldier fly larvae (BSFL) have shown great potential to serve as an alternative protein source for the livestock industry [74]. The black soldier fly larvae can be reared on a wide variety of decaying organic waste material, including agricultural products (fruits and vegetables) to food scraps, and manure from poultry, pigs, and cattle. Thus, the production of BSFL transforms waste products into a valuable protein source while simultaneously minimising the negative impact that may be caused by agricultural waste on the environment [75]. The CP content of BSFL ranges from 40 to 44% and is composed of exceptional quantities of valuable essential amino acids such as lysine (6% to 8% of the CP) [76]. Mbhele et al. [77] reported an optimum inclusion level of BSFL in Jumbo quail diets was 54 g/kg, which indicates that higher inclusion levels of this novel protein source could reduce feed intake and performance due to the presence of chitin, a polysaccharide of the arthropod’s exoskeleton [78].

### Use of alternative energy sources

Maize is the primary source of energy in poultry diets, which accounts the largest portion in poultry feeds [79]. Although maize is regarded as the main source of nutrition for a substantial proportion of people in tropical countries, a large quantity is redirected to other markets, such as biofuel, brewing, and starch production [80]. Consequently, this has led to shortage of maize available for livestock ration. In addition, insufficient production of this grain, which is further aggravated by the intense competition between man, industries, and livestock particularly in the semi-arid areas of the tropics has made poultry rations to be more expensive [81]. In response to this, animal nutritionists have identified and evaluated alternative energy sources that are less expensive, readily accessible, and locally available to mitigate the global food crisis. To reduce the over-reliance on maize grain as a major energy source in poultry, the use of low-tannin sorghum, millet varieties, wheat, cassava, and seaweeds has been studied [17, 82, 83].

#### Cassava

Cassava is a root crop tuber that is high in carbohydrates, minerals and vitamins B and C, and can be used as a substitute for corn in quail diets [84]. The plant originates from South America and was introduced to Africa in the sixteenth century to which it outcompeted some of the local crops as a staple crop in Africa [85]. Several studies have reported that 10–50% inclusion of dried cassava meal in place of maize in layer or broiler quail diets do not compromise the productive performance of the birds [84, 86]. Africa is the largest producer of cassava [87, 88], but this crop has not been adopted as a major feed ingredient in poultry diets because of the presence of anti-nutritional factors such as hydrocyanic acids [89]. However, standard processing methods of cassava which includes sun-drying, fermentation, boiling, soaking, and pelleting have been noted to improve the feed value of cassava meal by reducing HCN. Sun-drying alone was reported to eliminate almost 90% of cyanide content in cassava [90]. Similarly, complete replacement of maize was achieved when cassava was pre-treated with brewer’s yeast in broiler chicken diets [91].

#### Millet

Millet varieties (finger and pearl millets) are considered as valuable potential energy source for livestock feeding [92]. Millet varieties are generally drought-tolerant plants with the potential to thrive under harsh environmental conditions that are not suitable for corn and wheat [17]. Accordingly, pearl millet has been reported to produce yields that supersede that of other cereal grains when grown under poor environmental conditions, characterised by low soil fertility, heat stress, drought, and short growing season [93]. From a nutritional viewpoint, this grain is a valuable source of feed in quail diets as it is high in metabolisable energy (2900 kcal ME), which compares favourably well with that of maize (3330 kcal ME) [80] but, 40% richer in lysine and methionine compared to maize [94]. Millet’s drought and heat tolerance makes it a suitable crop that can be cultivated in tropical conditions, while its nutritional profile suggests it may be used in place of maize, particularly in quail feeds. The agro-ecological grain production methods of millet are well-suited to developing countries, and this grain has been found to promote comparable performance without compromising the health status of the birds [95]. Several studies found that pearl millet does not alter growth performance, intestinal development, and carcass traits in Japanese quail [81, 94]. Similarly, Masenya et al. [17] and Bulus et al. [96] have reported that millet grains can completely replace corn without adversely affecting the performance and health status of Jumbo quail and broiler chickens, respectively.

#### Sorghum

Sorghum is another potential energy source that has been used for many years in semi-arid or tropical regions to replace corn in poultry diets because it contains adequate nutritional composition, better harvest yield per area, and it is cheaper than maize [97]. The proximate analysis of sorghum compares favourably to that of maize, with sorghum having energy content (319 kcal/kg) that is marginally lower than that of corn, presumably because of the reduced oil resulting in slightly less energy value [98]. The competitive advantage of sorghum to maize is its resilience to harsh environmental conditions, being able to acclimatise in areas with marginal soils, high temperature, and low rainfall making it a hardier crop. For these reasons, sorghum remains largely a crop of small cultivators and is mostly consumed locally where it is cultivated [99]. Old sorghum varieties had considerable amounts of tannins, which limited its inclusion in quail diets because tannins are known to inhibit fibrolytic and proteolytic enzymes activities and reduce nutrient digestibility [100]. However, breeding of low-tannin sorghum varieties and hybrids had been developed and evaluated in poultry and swine feeds. For example, Younis [100] reported that 50% inclusion of low-tannin sorghum has no effect on nutrient utilisation and poses no toxicity to the quail. Moreover, Freitas et al. [101] observed that complete replacement of maize with sorghum had no dietary effect on performance traits and feed utilisation efficiency in Japanese quail. Likewise, Torres et al. [102] found that 100% replacement of maize with low-tannin sorghum grains did not affect quail and broiler performance, carcass yield and gut development.

### Use of potential natural additives

Many countries primarily those under the European Union have banned the use of antibiotic growth promoters in animal feeds due to various health concerns regarding antibiotic residues in animal products which may lead to the emergence of anti-microbial resistant pathogens [103]. This has prompted researchers to expedite the process of identifying and evaluating natural substances like medicinal plants that can act as an antioxidant, anti-microbial, anti-fungal, immune-modulatory, and anticoccidial properties [104]. There are several phytogenic plants (*Aloe vera*, *Moringa oleifera*, cinnamon, green tea, rosemary, and mint), waste by-products (apple, grape, pomegranate, citrus, and pawpaw pomaces), seeds (coriander and chickpea), and edible fungi (common mushroom) that have been reported to improve poultry performance by enhancing metabolic processes and efficiency of feed utilisation, while maintaining gut health and alleviating environmental stress [62, 103, 105]. These herbs or their extracts can be blended into a basal quail diet or administered orally through drinking water and have been reported to enhance intestinal microflora by proliferating beneficial bacteria in poultry birds [27, 104].

Herbs are widely used in the feed as preservatives against oxidative deterioration during storage [106]. In addition, herbs and their extracts have multiple purposes in the body of birds such as enhancing digestive tract activity, as well as exhibiting anti-inflammatory, anti-oxidative, and anti-microbial properties [105]. For instance, *Moringa oleifera* leaf meal is an exceptional source of antioxidant compounds like ascorbic acid, flavonoids, phenolics, and carotenoids, which have prebiotic properties as well as antioxidant phytochemicals like chlorogenic acid and caffeic acid [107]. Elkloub et al. [108] reported that supplementing basal diets with 2 g/kg of *M*. *oleifera* improve performance, immune organs, and blood constituents in Japanese quail. Mulaudzi et al. [27] also found that the addition of 25 g/kg *Moringa oleifera* leaf meal increases weight gain in adult female Japanese quail birds without affecting their physiological and meat quality responses. According to Manafi et al. [109], the use of photo-molecules bioactive compounds (allicin, peppermint, thymol, and carvacrol) and organic acids (propionic acid and fumaric acid) in Japanese quail resulted in similar feed consumption while promoting higher egg production due to utilisation of feed more efficiently than those raised on diets containing antibiotic growth promoter. Similar findings were described by Güler et al. [103], who noted an improvement in feed intake, weight gain and feed conversion ratio in quail reared on coriander (*Coriandrum sativum*)-containing feed. These improvements have been associated with the appetising effect of essential oils and their main component such as linalool in coriander seeds, which stimulates the digestive process [110]. Therefore, supplementing quail rations with locally available and naturally growing phytogenic herbs would aid digestion, improve performance and health of the birds.

## Roles of farmers and extension services on consumer awareness

For the South African quail sector to stabilise and flourish, quail farmers would need to first promote the potential benefits of quail meat and eggs. Quail egg producers should be alert that there are consumers in many communities who still refrain from egg consumption due to perceived high cholesterol content, which is associated with coronary heart diseases [49]. For this group, farmers through extension officers will have to double their efforts in educating consumers about the benefits of quail egg consumption. Ogunwole et al. [111] noted that socio-economic characteristics influence the decisions on whether to consume quail eggs or not. Thus, there is a dire need for extension services (especially from local government) to educate the public about the potential benefits of quail products, especially those who are still sceptical about consuming quail eggs or meat.

In light of the current COVID-19 pandemic that has engulfed the world, emerging quail farmers could benefit from financial assistance from government agencies to boost their enterprises. Being a relatively new poultry sector in South Africa, coupled with movement restrictions brought by the COVID-19 pandemic since early 2020, most government advisory services have been impacted and, in most cases, unable to work at full capacity. Compounding these difficulties is the shortage of extension officers who specialise in quail advisory services. Apart from verbal interactions, most South African extension officers can prepare free manuals and pamphlets on quail rearing, management strategies or manuals that deal with health problems that may arise in quail farming.

Quail farming has a huge potential to grow in South Africa [16], however, most farmers still do not achieve optimum production due to a lack of professional training, failure to meet extension officers and high feed costs. Most small-scale farmers in South Africa are located in remote rural or mountainous areas and logistically it becomes complicated to be located by the generally urban-based government extension officers [112]. The emergence of COVID-19 further intensified this problem as fewer services from government are provided to the farmers. This situation is prohibitive to quail farming, while reducing the ability to contribute to food and nutrition security.

In South Africa, most state extension workers are deployed as generalists at provincial level, serving large- and small-scale farmers. By the end of 2012, government extensionist-to-client ratio in South Africa averaged 1:1034 against 1:171 for the private sector [113]. The few extension agents in relation to the number of farmers is at the centre of factors that leads to limited access to extension services in the poultry sector. Oakley and Garforth [114], lamented the imbalanced farmer-extension officer ratio in developing countries as, in most cases, extension services are directed to resourceful farmers who are likely to adopt modernised innovations in well-established areas. Unfortunately, rural farmers do not have the privilege to gain full access to agricultural information from other sources besides government extension services. Similarly, rural quail farmers are also constrained from getting useful information from extension officers due to their level of education, which then limit active participation in training sessions that uses a lot of written material [115]. To fully disseminate information to prospective quail farmers, government and its stakeholders should prioritise increasing the number of skilled extension officers to improve the extensionist-to-client ratio. There is also a need for regular face-to-face interaction with extension agents, and other information and communication technologies (ICTs) based online interaction platforms [116].

## Possible marketing strategies for quail producers

The South African quail sector is still in its infancy stages, thus more efforts should be directed towards building a long-lasting and effective market base. For quail products to gain popularity to broader consumers in South Africa, both the farmers and extension officers should exert more emphasis on the distribution of information targeting local based consumers [117]. This would involve the utilisation of local media outlets such as newspaper adverts, community radio stations and distribution of flyers at local shopping centres. These activities are mostly cost-effective, and in some cases, they offer free marketing platforms that may drive the consumers to be aware of quail farming and its products [118]. To fast track the distribution of quail products to local retail stores, intermediary mechanism such as the utilisation of the salesmen (agents) is an important form of getting the products and information out to the consumers as these agents would purchase bulk of quail products and sell to the wholesalers and retailers [119]. Social media platforms such as Twitter, Facebook, WhatsApp to mention but a few can be the fastest and most economical ways in which quail products can be advertised. Although these platforms, may not reach the rural citizenry, road signs and boards can be considered in rural areas.

## Building a local market niche area for the quail sector

Like in any business enterprise, product marketing should always be one of first considerations. The South African poultry industry in a global market and, in the last decade, local producers have struggled to compete due to rising feed costs and the recent global crisis brought by COVID-19 that have stagnated the export market [120]. All these challenges necessitate the need to develop a solid local market base to limit the reliance of the industry on international markets. Quail farming presents an opportunity for South African poultry farmers to develop their own niche market, considering that natural pandemics such as COVID-19 will continue to be a stumbling block especially for export markets. A cost-effective quail farming system could, in a long run, be a solution and ultimately persuade many local farmers to intensify production. To fully appreciate the benefits of quail farming, the following marketing strategies can be adopted:

i. Farmers can join legally recognised poultry regulatory bodies such as the South Africa Poultry Association (SAPA), where they would be assisted to make valuable contacts with other role players (feed producers) and customers.
ii. Attend government-based short learning programs, local poultry conferences, meetings, and workshops to empower their marketing strategy by connecting them with broader audiences, including potential investors in the quail farming enterprises.
iii. Invest in contemporary advertising strategies that are, nowadays, channelled through social media platforms. These adverts should be clear, attractive, and concise to appeal to diverse consumer groups, both in rural and urban communities (restaurants and hotels).
iv. Keep a clean and bio-secure quail farm because prospective customers’ judge farm produce based on appearance and the physical outlook of the farm.
v. Prioritise customer’ satisfaction and respond to all queries regarding quail products with enthusiasm, as it is one of the best marketing strategies in the world to grow clientele.
vi. Guide prospective or emerging farmers on the advantages of quail products without running down fellow competitors because farmers need one another to grow their businesses (cooperatives) and popularity of their products.

## Conclusions

The nutraceutical benefits that come with quail products can contribute to food and nutrition security in South Africa. However, sustainable intensification of quail is largely constrained by high production costs, while quail sales are disadvantaged by the low popularity and demand of the birds, which continue to exclude quail products from the markets. Efforts should be made to overcome these challenges by using nutritional solutions and marketing strategies that would promote a sustainable and profitable quail farming business. Future studies can be designed to analyse consumers’ preferences that influence the consumption of quail meat and eggs to identify their needs and subsequently increase quail demand in South Africa.

## Data Availability

Not applicable.
